# Neutrophil extracellular traps in retrieved thrombi and functional outcome after stroke thrombectomy

**DOI:** 10.3389/fneur.2026.1779666

**Published:** 2026-04-22

**Authors:** Lizhang Chen, Fayun Hu, Hongbo Zheng, Yanbo Li, Li He

**Affiliations:** 1 West China Hospital, Sichuan University, Chengdu, China; 2 Department of Neurology, West China Tianfu Hospital, Sichuan University, Chengdu, China

**Keywords:** acute ischemic stroke, biomarker, mechanical thrombectomy, neutrophil extracellular traps, prognosis, Thrombus composition

## Abstract

**Objective:**

To investigate the association between neutrophil extracellular traps (NETs) content in retrieved thrombi and 90-day functional outcomes in patients with acute ischemic stroke (AIS) secondary to anterior circulation large vessel occlusion (LVO) undergoing mechanical thrombectomy (MT).

**Methods:**

This retrospective study analyzed data from a prospectively maintained cohort of 129 consecutive AIS patients who underwent MT at West China Hospital between January 2023 and October 2024. Retrieved thrombi were stained for citrullinated histone H3 (CitH3) and myeloperoxidase (MPO) using immunofluorescence, and the percentage of positive area was quantified to represent NETs content (% area). Patients were dichotomized into high-NETs and low-NETs groups based on an optimal cutoff determined by receiver operating characteristic (ROC) curve analysis for predicting poor outcome (modified Rankin Scale [mRS] score 3–6). The primary endpoint was functional independence (mRS 0–2) at 90 days. Multivariable logistic regression was employed to identify independent predictors of outcome.

**Results:**

The ROC analysis yielded an AUC of 0.644. The optimal cutoff value of defining high NETs content was 2.01%. Among 129 patients (mean age 67.9 years, 48.8% male), 88 (68.2%) were in the high-NETs group. Baseline characteristics were largely comparable, though the high-NETs group had significantly higher admission blood glucose (7.9 vs. 6.7 mmol/L, *p* = 0.009) and a greater prevalence of atrial fibrillation (65.9% vs. 36.6%, *p* = 0.002). Despite achieving similarly high rates of successful recanalization (mTICI 2b-3) in both groups (96.6% vs. 97.6%, *p* = 0.99), patients in the high-NETs group had a significantly lower rate of 90-day functional independence (35.2% vs. 68.3%, *p* < 0.001). After adjusting for confounding variables, high thrombus NETs content remained a robust independent predictor of a lower likelihood of achieving a good outcome (adjusted Odds Ratio [aOR] = 0.60, 95% CI 0.43–0.84, *p* = 0.003), along with higher baseline NIHSS (aOR = 0.90, 95% CI 0.84–0.97, *p* = 0.007) and an increased number of retrieval attempts (aOR = 0.66, 95% CI 0.46–0.96, *p* = 0.028).

**Conclusion:**

Quantitative assessment of NETs in retrieved thrombi may serve as an accessible biomarker to identify patients at high risk of poor functional recovery despite successful recanalization. However, the predictive performance was only moderate (ROC AUC = 0.644). Moreover, infarct core volume was not assessed and thus not incorporated into the statistical models, which may limit interpretation and generalizability. Prospective multicenter validation and studies incorporating infarct core volume are warranted, together with exploration of NET-targeted adjunctive therapies.

## Introduction

Acute ischemic stroke (AIS) remains a leading cause of mortality and long-term disability worldwide (1, 2). The advent of mechanical thrombectomy (MT) for large vessel occlusion (LVO) has revolutionized acute stroke care, substantially improving rates of vessel recanalization and functional independence (3–7). However, a significant proportion of patients, estimated at nearly 50%, fail to achieve a good functional outcome despite successful and timely reperfusion (3–7). This phenomenon highlights a critical knowledge gap and underscores the influence of factors beyond simple vessel patency.

Thrombus composition is increasingly recognized as a key determinant of both treatment response and clinical outcome. The cellular and molecular makeup of a thrombus influences its mechanical properties, such as stiffness and friability, which can impact the efficacy of MT (8–11). Recently, neutrophil extracellular traps (NETs) have emerged as a critical component of arterial thrombi (12, 13), playing a role in the property of stiffness and friability. NETs are web-like structures composed of decondensed chromatin, histones, and granular proteins, released by activated neutrophils during a process termed NETosis (14, 15). By providing a robust scaffold for fibrin deposition and platelet aggregation, NETs enhance thrombus stability and increase resistance to both enzymatic thrombolysis (16, 17) and mechanical disruption (8). Growing evidence suggests a strong correlation between NETs levels and poor clinical prognosis in ischemic stroke (18–21). Elevated levels of circulating NETs markers have been linked to greater stroke severity and worse functional outcomes (20, 21). To balance NETs formation and clearance, thereby mitigating their pathogenic side effects is of great importance in thrombus intervention. Furthermore, histological studies have confirmed the presence of NETs within cerebral thrombi (22, 23), where their abundance has been associated with increased procedural difficulty and a higher number of retrieval attempts (8, 9, 24).

Despite these advances, the direct prognostic impact of intra-thrombus NETs content on long-term functional outcomes after MT, independent of recanalization success, remains inadequately characterized (25, 26). It remains unclear whether the detrimental effects of NETs are confined to procedural challenges or extend to post-recanalization pathophysiology. Furthermore, as the contribution of NETs to thrombus stability and treatment resistance can vary significantly under different circumstances, the potential value of a personalized anti-NETs strategy lies in stratifying patients based on their thrombus NET burden or specific NETosis pathway, thereby guiding targeted adjunctive therapies (e.g., DNase, PAD4 inhibitors) to degrade NETs or prevent their formation, with the goal of overcoming treatment resistance, improving revascularization success, and ultimately enhancing clinical outcomes. Therefore, this study aimed to investigate the association between the quantified NETs content in retrieved thrombi and 90-day functional outcomes in a well-defined cohort of AIS patients undergoing MT, while rigorously controlling for established clinical, imaging, and procedural predictors. We hypothesized that a higher burden of NETs within the thrombus would be independently associated with a lower probability of achieving functional independence at 90 days.

## Methods

### Study design and patient population

This study was a retrospective analysis of data from a prospectively maintained institutional stroke registry. We included 129 consecutive patients with AIS due to an anterior circulation LVO (terminal internal carotid artery or middle cerebral artery M1/M2 segments) who underwent MT at the Department of Neurology, West China Hospital of Sichuan University, between January 2023 and October 2024. The study was conducted in accordance with the Declaration of Helsinki and was approved by the Institutional Review Board of West China Hospital. Due to the retrospective nature of the analysis of anonymized data, a waiver of individual informed consent was granted.

Inclusion criteria were: (1) age ≥ 18 years; (2) symptom onset within 24 h; (3) confirmed LVO on computed tomography angiography (CTA) or digital subtraction angiography (DSA); (4) pre-stroke modified Rankin Scale (mRS) score ≤1; (5) baseline National Institutes of Health Stroke Scale (NIHSS) score >6; and (6) successful retrieval of a thrombus sample of sufficient size for histological analysis. Exclusion criteria included: (1) primary treatment with angioplasty and/or stenting for underlying intracranial atherosclerotic occlusion without thrombectomy; (2) posterior circulation occlusion; (3) incomplete 90-day follow-up data; or (4) severe, life-limiting systemic comorbidities.

### Thrombus collection and processing

Thrombectomy procedures were performed by experienced neurointerventionalists using stent retrievers, direct aspiration, or a combined technique, at the discretion of the operator. Upon retrieval, thrombus material was immediately rinsed with saline. A representative portion was fixed in 4% neutral-buffered formalin for 24 h, processed through a standard paraffin-embedding protocol, and sectioned for subsequent analysis. The remaining portion was flash-frozen in liquid nitrogen and stored at −80 °C for future research.

### Immunofluorescence staining and NETs quantification

Formalin-fixed, paraffin-embedded thrombus sections (4-μm thick) were mounted on charged slides. Sections underwent deparaffinization, rehydration, and heat-induced antigen retrieval. After blocking with 5% bovine serum albumin, sections were incubated overnight at 4 °C with a cocktail of primary antibodies: rabbit anti-Citrullinated Histone H3 (CitH3, a specific marker of NETosis; 1:100) and mouse anti-Myeloperoxidase (MPO, an abundant neutrophil enzyme; 1:400). Following washes, sections were incubated for 1 h at room temperature with corresponding Alexa Fluor 594-conjugated anti-rabbit and Alexa Fluor 488-conjugated anti-mouse secondary antibodies. Nuclei were counterstained with 4′,6-diamidino-2-phenylindole (DAPI).

Whole-slide images were acquired using an Olympus VS200 scanning system. NETs were defined as extracellular, web-like structures exhibiting co-localizing of CitH3 (red) and MPO (green) signals, often associated with decondensed DAPI (blue) staining. Using Halo image analysis software (Indica Labs), a custom algorithm was developed to quantify the total tissue area and the area positive for NETs (co-localized CitH3/MPO signal). The NETs content was expressed as the percentage of the NETs-positive area relative to the total tissue area. We analyzed 5 randomly selected non-overlapping sections in each intact thrombus, and the average NETs positive area across all 5 sections was defined as the final NET burden value for each sample. Image analysis was performed by two independent investigators blinded to all clinical and outcome data.

### Group stratification

To facilitate clinical translation, NETs burden was categorized into high and low groups based on a receiver operating characteristic (ROC)-determined cutoff. This binary framework aligns with clinical decision-making paradigms, increases analytical robustness, and anticipates the format of future point-of-care NETs assays. To establish an objective threshold for NETs content, the ROC curve was generated using the 90-day mRS score (dichotomized as good outcome [mRS 0–2] vs. poor outcome [mRS 3–6]) as the state variable. The Youden index was used to determine the optimal cutoff value of NETs percentage that maximized the sum of sensitivity and specificity. Patients were subsequently stratified into a high-NETs group (NETs content ≥ cutoff) and a low-NETs group (NETs content < cutoff).

### Data collection and outcomes

Comprehensive clinical, procedural, and imaging data were extracted from the electronic medical records. This included: *Baseline Data*: Age, sex, vascular risk factors, pre-admission medications (statins, antiplatelets, anticoagulants), admission blood glucose, and etiology of stroke based on the Trial of Org 10,172 in Acute Stroke Treatment (TOAST) classification. *Clinical Severity*: NIHSS scores at baseline and 24 h post-procedure; pre-stroke mRS. *Imaging Data*: Alberta Stroke Program Early CT Score (ASPECTS), presence of a hyperdense artery sign, and thrombus density (Hounsfield units) on non-contrast CT. *Procedural Data*: Onset-to-groin puncture time, procedure duration, number of retrieval attempts, first-pass effect (mTICI 3 after a single attempt), and final recanalization status, assessed using the modified Thrombolysis in Cerebral Infarction (mTICI) scale. Successful recanalization was defined as mTICI 2b-3. *Outcomes*: The primary outcome was functional independence at 90 days, defined as an mRS score of 0–2. Secondary outcomes included symptomatic intracranial hemorrhage (sICH) according to the ECASS II criteria and early neurological improvement (decrease of ≥4 points in NIHSS at 24 h).

### Statistical analysis

Continuous variables were tested for normality using the Shapiro–Wilk test. Normally distributed data were presented as mean ± standard deviation (SD) and compared using Student’s *t*-test. Non-normally distributed data were presented as median (interquartile range, IQR) and compared using the Mann–Whitney U test. Categorical variables were presented as counts (percentages) and compared using the Chi-square test or Fisher’s exact test, as appropriate.

To identify independent predictors of 90-day functional independence (mRS 0–2), a binary logistic regression analysis was performed. Variables with a *p*-value < 0.10 in the univariate analysis, along with clinically relevant variables (e.g., age), were entered into a multivariable logistic regression model using a backward stepwise method. Results were reported as odds ratios (OR) with 95% confidence intervals (CI). A two-sided *p*-value < 0.05 was considered statistically significant. All analyses were performed using SPSS software (Version 26.0, IBM Corp).

## Results

### Patient cohort and NETs stratification

**Figure 1:**
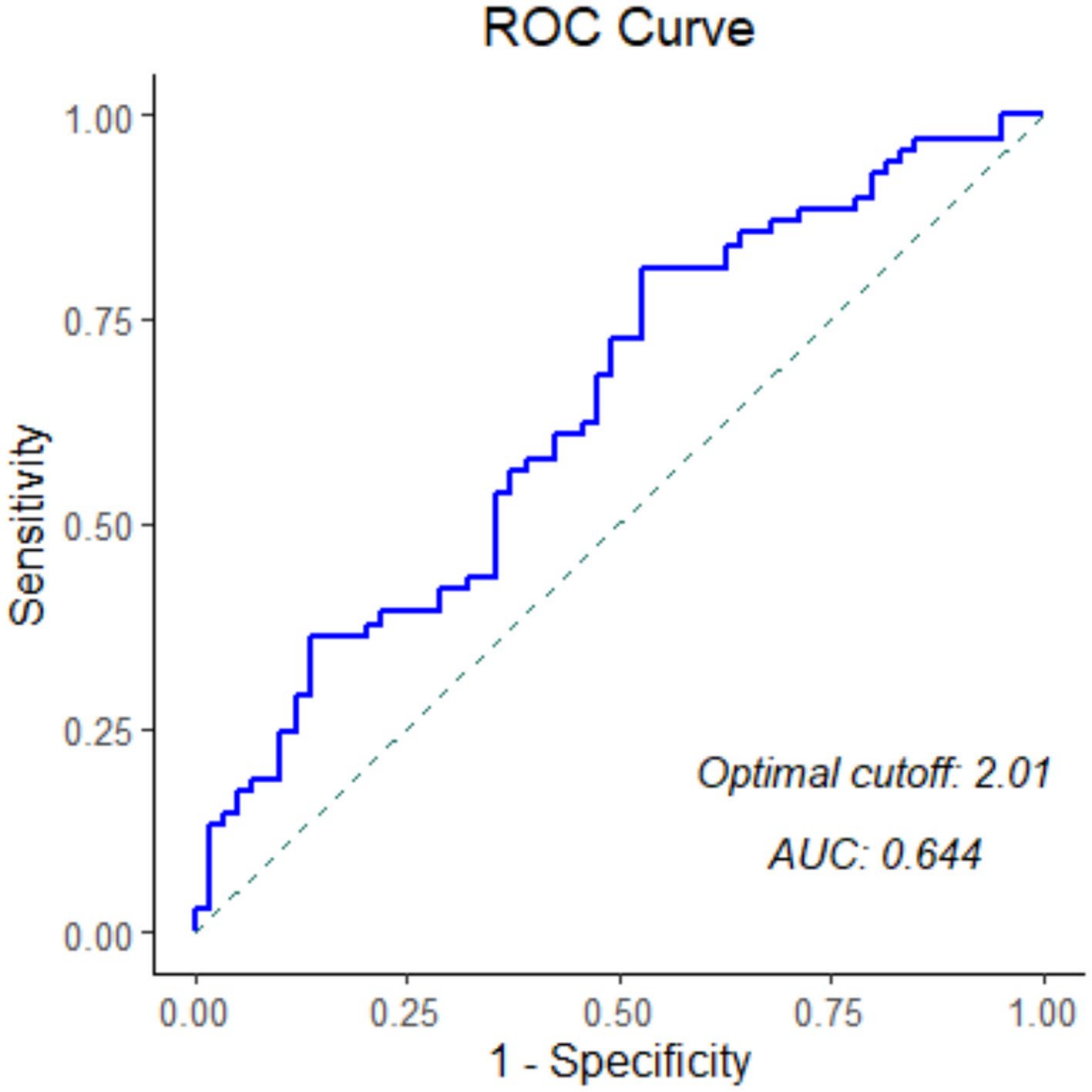
Receiver operating characteristic (ROC) curve of NETs expression. The x-axis represents 1 − specificity, and the y-axis represents sensitivity. The blue line indicates the ROC curve constructed according to NETs expression. The area under the curve (AUC) was 0.644, and the optimal cutoff value was 2.01.

**Figure 2:**
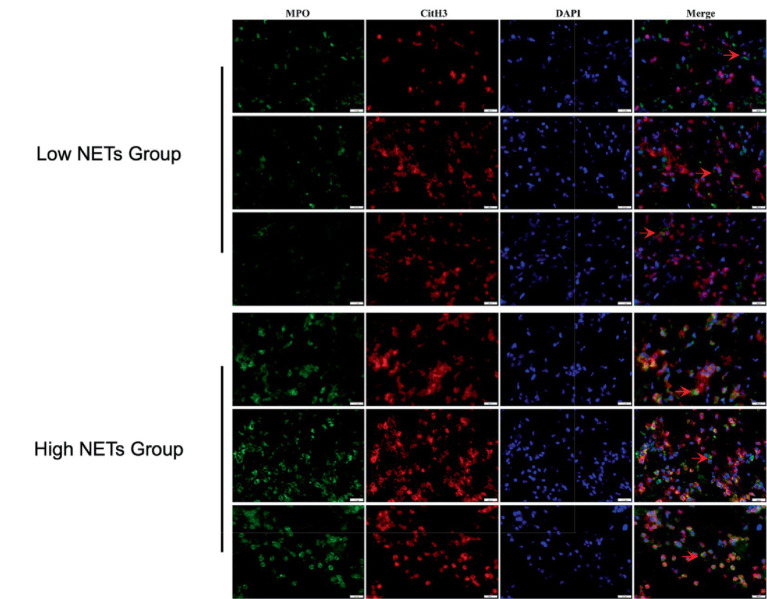
Representative immunofluorescence staining images of high and low NETs groups. Images were captured at 400× magnification. Three representative cases were selected from each group for presentation. Green fluorescence indicates MPO-positive staining, red fluorescence indicates CitH3 staining, blue fluorescence indicates DAPI-labelled nuclei, and “Merge” shows the overlay of all channels. The white bar in the lower right corner represents 20 μm.

A total of 129 patients met the inclusion criteria and were included in the final analysis. The mean age was 67.9 ± 15.8 years, and 63 (48.8%) were male. The median baseline NIHSS was 14 (IQR 11–18). The ROC analysis for thrombus NETs content predicting 90-day mRS 0–2 yielded an area under the curve (AUC) of 0.644 (95% CI 0.547–0.741, *p* < 0.001), with a moderately optimal cutoff value of 2.01% (Figure 1). Based on this threshold, 88 patients (68.2%) were assigned to the high-NETs group, and 41 patients (31.8%) to the low-NETs group. Representative immunofluorescence images are shown in Figure 2.

### Baseline and procedural characteristics

Baseline characteristics are detailed in Table 1. The two groups were well-matched in terms of age, sex, pre-stroke mRS, baseline NIHSS, and most vascular risk factors. However, patients in the high-NETs group presented with significantly higher admission blood glucose levels (7.9 ± 2.9 vs. 6.7 ± 1.5 mmol/L, *p* = 0.009) and had a significantly higher prevalence of atrial fibrillation (65.9% vs. 36.6%, *p* = 0.002), resulting in a greater proportion of cardioembolic strokes based on TOAST classification.

**Table 1:** Baseline characteristics of patients stratified by thrombus NETs content.

Characteristic	Total (*n* = 129)	High NETs group (*n* = 88)	Low NETs group (*n* = 41)	*p*-value
Age, mean ± SD, years	67.9 ± 15.8	69.3 ± 15.3	65.0 ± 16.4	0.147
Male, *n* (%)	63 (48.8)	45 (51.1)	18 (43.9)	0.444
Admission glucose, mmol/L	7.6 ± 2.6	7.9 ± 2.9	6.7 ± 1.5	0.009
NETs, %, mean ± SD	2.8 ± 1.7	3.6 ± 1.6	1.3 ± 0.4	<0.001
History				
Smoking, *n* (%)	21 (16.3)	11 (12.5)	10 (24.4)	0.089
Alcohol, *n* (%)	24 (18.6)	15 (17.0)	9 (22.0)	0.505
Hypertension, *n* (%)	67 (51.9)	49 (55.7)	18 (43.9)	0.212
Diabetes mellitus, *n* (%)	24 (18.6)	19 (21.6)	5 (12.2)	0.202
Atrial fibrillation, *n* (%)	73 (56.6)	58 (65.9)	15 (36.6)	0.002
Previous stroke, *n* (%)	18 (14.0)	13 (14.8)	5 (12.2)	0.694
Pre-admission medication				
Statin, *n* (%)	21 (16.3)	16 (18.1)	6 (14.6)	0.337
Antiplatelet, *n* (%)	18 (14.0)	12 (13.6)	6 (14.6)	0.879
Anticoagulants, *n* (%)	31 (24.0)	21 (23.9)	10 (24.4)	0.948
Pre-stroke mRS, median (IQR)	0 (0–4)	0 (0–0)	0 (0–0)	0.340
Baseline NIHSS, median (IQR)	14 (11–18)	14 (11–18)	13 (10–18)	0.437
Baseline ASPECTS, median (IQR)	7 (6–8)	7 (6–8)	7 (6–9)	0.473

Procedural and imaging characteristics are presented in Table 2. There were no significant differences between the groups regarding onset-to-treatment times, rates of bridging intravenous thrombolysis, procedure duration, or the number of retrieval attempts. Notebly, the rate of successful recanalization (mTICI 2b-3) was exceptionally high and comparable between groups (96.6% in high-NETs vs. 97.6% in low-NETs, *p* = 0.99). Rates of first-pass effect and periprocedural complications, including sICH, were also similar. Despite similar recanalization success, the median 24-h NIHSS score was significantly higher in the high-NETs group compared to the low-NETs group (13 [IQR 8–19] vs. 9 [IQR 5–13], *p* = 0.036).

**Table 2:** Effect of NETs level on procedure-related parameters and imaging characteristics in AIS patients.

Characteristic	Total (*n* = 129)	High NETs group (*n* = 88)	Low NETs group (*n* = 41)	*p*-value
24 h NIHSS, median (IQR)	12 (6–18)	13 (6–20)	9 (4–17)	0.036
Onset-to-puncture time, min, median (IQR)	240 (150–420)	240 (120–405)	300 (180–320)	0.492
IV thrombolysis, *n* (%)	18 (14.0)	9 (10.2)	9 (22.0)	0.074
Procedure-related parameters				
Operation time, min, median (IQR)	53 (34–80)	52 (34–75)	70 (43–91.5)	0.061
Number of passes, median (IQR)	1 (0–2)	1 (0–2)	1 (0–3)	0.733
Stent retriever passes, median (IQR)	1 (1–2)	1 (1–2)	1 (0–3)	0.493
Aspiration passes, median (IQR)	1 (1–2)	1 (1–2)	1 (1–2.5)	0.290
Imaging characteristics				
MCA tortuosity, *n* (%)	10 (7.8)	7 (8.0)	3 (7.3)	0.900
Hyperdense artery sign, *n* (%)	65 (50.4)	42 (47.7)	23 (56.1)	0.669

### Functional outcomes

A striking divergence in 90-day functional outcomes was observed between the two groups (Table 3). The rate of functional independence (mRS 0–2) was nearly halved in the high-NETs group compared to the low-NETs group (35.2% vs. 68.3%, *p* < 0.001). Conversely, the rate of severe disability or death (mRS 4–6) was significantly higher in the high-NETs group (50.0% vs. 26.8%, *p* = 0.013). The overall distribution of 90-day mRS scores showed a significant shift towards worse outcomes in the high-NETs cohort (*p* = 0.009 for trend).

**Table 3:** Effect of NETs level on vascular recanalization and neurological outcomes in AIS patients.

Variable	Total (*n* = 129)	High NETs group (*n* = 88)	Low NETs group (*n* = 41)	*p*-value
Vascular recanalization				
Successful recanalization, *n* (%)	125 (96.9)	86 (97.7)	39 (95.1)	0.427
First-pass successful recanalization, *n* (%)	34 (26.4)	22 (25.0)	12 (29.3)	0.608
Symptomatic intracranial hemorrhage, *n* (%)	14 (10.9)	11 (12.5)	3 (7.3)	0.378
Any intracranial hemorrhage, *n* (%)	29 (22.5)	20 (22.7)	9 (22.0)	0.922
Early neurological outcome				
Early neurological improvement, *n* (%)	21 (16.3)	11 (12.5)	10 (24.4)	0.089
Early neurological deterioration, *n* (%)	20 (15.5)	15 (17.0)	5 (12.2)	0.479
90-day functional outcome				
90-day mRS, median (IQR)	3 (1–6)	3 (1–6)	2 (1–4)	0.009
90-day mRS 0–2, *n* (%)	59 (45.7)	31 (35.2)	28 (68.3)	<0.001
90-day mRS 4–6, *n* (%)	55 (42.6)	44 (50.0)	11 (26.8)	0.013

### Predictors of 90-day functional outcome

In the multivariable logistic regression model (Table 4), after adjusting for age, atrial fibrillation, admission glucose, baseline NIHSS, and number of retrieval attempts, high thrombus NETs content remained a strong and independent negative predictor of a good 90-day outcome (adjusted OR = 0.60, 95% CI 0.43–0.84, *p* = 0.003). Other independent predictors of a lower likelihood of good outcome were a higher baseline NIHSS score (aOR = 0.90, 95% CI 0.84–0.97, *p* = 0.007) and a greater number of retrieval attempts (aOR = 0.66, 95% CI 0.46–0.96, *p* = 0.028). Pre-admission use of anticoagulants was independently associated with a higher chance of good outcome (aOR = 3.20, 95% CI 1.09–9.35, *p* = 0.033).

**Table 4:** Factors associated with 90-day good outcome (mRS 0–2) in AIS patients.

Variable	Univariate OR (95% CI)	*p*-value	Multivariate OR (95% CI)	*p*-value
Age, years	0.97 (0.94–0.99)	0.006	0.99 (0.95–1.02)	0.401
Male sex	1.61 (0.80–3.25)	0.179	—	—
Admission glucose, mmol/L	0.80 (0.67–0.95)	0.010	0.85 (0.69–1.03)	0.107
NETs, %	0.67 (0.51–0.88)	0.004	0.60 (0.43–0.84)	0.003
Hypertension	0.49 (0.24–0.99)	0.047	1.28 (0.47–3.46)	0.715
Diabetes mellitus	0.33 (0.12–0.89)	0.028	0.50 (0.13–1.80)	0.285
Atrial fibrillation	0.39 (0.19–0.79)	0.009	0.65 (0.23–1.86)	0.424
Previous stroke	0.55 (0.19–1.56)	0.260	—	—
Statin use	1.15 (0.45–2.95)	0.772	—	—
Antiplatelet use	1.38 (0.50–3.83)	0.531	—	—
Anticoagulant use	2.30 (1.00–5.25)	0.049	3.20 (1.09–9.35)	0.033
Baseline NIHSS score	0.90 (0.84–0.96)	0.001	0.90 (0.84–0.97)	0.007
IV thrombolysis	2.06 (0.74–5.71)	0.164	—	—
Number of passes	0.75 (0.56–1.00)	0.052	0.66 (0.46–0.96)	0.028
Successful recanalization	0.00 (0.00–0.00)	0.999	—	—
MCA tortuosity	0.12 (0.01–0.95)	0.045	0.13 (0.12–1.26)	0.079
Hyperdense artery sign	0.97 (0.48–1.93)	0.924	—	—

## Discussion

In this comprehensive analysis of patients with AIS undergoing MT, we identified a high burden of NETs within retrieved thrombi as a potential independent predictor of poor 90-day functional outcome. This association remained relatively robust after rigorous adjustment for key clinical and procedural confounders, including baseline stroke severity and recanalization status. These findings point to the possibility that the biological composition of the thrombus, particularly its NETs load, could influence stroke recovery, and that this influence, at least in part, independent of the technical success of revascularization.

Links were observed between high-NET thrombi, admission hyperglycemia, and atrial fibrillation which represent a valuable piece of clinical context for our observations, though their causal nature requires further investigation. Hyperglycemia is a well-established inducer of NETosis (27, 28), potentially via pathways like oxidative stress, thereby linking a common metabolic derangement in acute stroke to a specific prothrombotic mechanism. Similarly, the pro-inflammatory and prothrombotic milieu created by atrial fibrillation is conducive to neutrophil activation and NET formation (29–32). Our data suggest that these clinical risk factors may converge on the NETosis pathway, leading to the formation of NET-rich thrombi that are intrinsically associated with worse outcomes. Nevertheless, as a retrospective study, our findings establish an association but preclude a causal inference as to whether hyperglycemia or atrial fibrillation directly triggered the formation of the NET-rich thrombus in the index stroke event. Future prospective studies are warranted to explore the dynamic and causal relationships between these risk factors and thrombus NET content (25, 26). Recent studies have confirmed another factor, obesity, tends to promote the formation of NETs *in vivo*, which partially explains the association between obesity and chronic inflammation as well as metabolic diseases (such as insulin resistance and atherosclerosis). As a modifiable risk factor for cerebrovascular disease, obesity and NETs—as a potential mediator of its impact on cerebrovascular pathology—represent a critical link. However, due to severe neurological deficits of patients, body weight data could not be fully collected, which prevented the inclusion of this factor in our analysis. Additionally, given that obese patients may exhibit lower mortality and fewer adverse outcomes after thrombectomy compared to normal-weight patients, the relationship between obesity, thrombectomy, and neurological functional outcomes in cerebrovascular disease is complex. We intend to further investigate this issue in future studies.

A pivotal finding of our study is that the detrimental impact of NETs on functional outcome persisted despite the high rate of successful recanalization in our cohort. This finding provides compelling evidence that their harmful effects extend beyond merely increasing the difficulty of thrombectomy (8, 24), and are likely mediated through post-recanalization pathophysiology. We propose several plausible and non-mutually exclusive mechanisms: First, NET-rich thrombi may be more friable during retrieval, leading to the shedding of NET-laden micro-emboli that occlude the distal microcirculation. This could contribute to the “no-reflow” phenomenon, thereby compromising tissue reperfusion at the capillary level. Second, NET components, particularly extracellular DNA and histones, are potent pro-inflammatory and cytotoxic molecules. Upon restoration of blood flow, their release into the cerebral microenvironment could exacerbate ischemia–reperfusion injury, disrupting the integrity of the blood–brain barrier, promoting vasogenic edema, and exerting direct neurotoxicity. While our study could not directly substantiate these mechanisms, we observed that patients in the high-NET group had significantly higher NIHSS scores at 24 h despite comparable recanalization rates, a finding consistent with more severe parenchymal injury that lends indirect clinical support to these hypotheses. In contrast to some prior studies that linked NETs to procedural difficulties (8, 24), we did not observe a significant association between NET content and the number of thrombectomy passes or first-pass success rate. This could be attributable to the high overall efficacy of the modern thrombectomy devices used in our cohort, or it may indicate that while NETs confer structural stability to the thrombus, this stability does not always translate into a measurable increase in retrieval attempts with current technologies. Nonetheless, the number of retrieval passes itself remained an independent predictor of outcome, reinforcing the established notion that procedural efficiency is paramount for patient recovery.

A key clinical implication of our research is the potential of quantifying NETs in retrieved thrombi as a novel prognostic biomarker. The analysis of thrombectomy material, available immediately post-procedure, could be used to refine current prognostic models that rely on clinical and imaging data, thereby enabling earlier and more accurate risk stratification. Furthermore, identifying patients with NET-rich thrombi could pave the way for personalized stroke therapy in the future (23, 33, 34). Such patients would be ideal candidates for testing NET-targeted adjuvant therapies, such as DNase to degrade the DNA backbone, inhibitors of PAD4 (a key enzyme in NETosis), or agents that neutralize cytotoxic histones. Naturally, the safety and efficacy of these potential therapies require rigorous preclinical evaluation before their translation into clinical trials (17, 35–38).

Our results align with and significantly extend the findings of the SVIN study (39), which demonstrated that a high NET burden within thrombi is associated with worse post-thrombectomy outcomes. This moves beyond correlation to suggest a more direct, potentially causal, role for NETs in the pathophysiology of poor recovery, elevating the thrombus NET load from a simple biomarker of association to a candidate biomarker of independent risk, in spite of successful recanalization. The clinical findings from our study gain profound biological plausibility when integrated with the mechanistic insights from the JCI study (40). It posits that the NET burden quantified in the thrombus is not just a passive marker but a quantifiable snapshot of a patient’s propensity for severe ischemia–reperfusion injury, explaining why some patients fare poorly despite technically successful recanalization.

Besides, accumulating evidence suggests that NETs play a significant pathogenic and prognostic role across acute arterial thrombotic syndromes, including ST-elevation myocardial infarction (STEMI). The data have demonstrated that patients with STEMI and a higher NET burden exhibit more adverse angiographic characteristics, higher rates of stress-induced hyperglycemia, and worse cardiovascular outcomes (41). The studies including our study raise a novel point on NETs that it may represent a shared pathogenic driver of thrombo-inflammation underlying poor outcomes across different acute ischemic vascular territories, thereby strengthening the translational potential of anti-NETs therapy as a universal intervention for acute arterial thrombosis(beyond stroke alone).

### Limitations

Our study is subject to several key limitations. The single-center, retrospective design introduces inherent risks of selection and referral bias. Infarct core volume, collateral status, and post-procedural blood pressure management were also unavailable and absent in the confounder analysis, due to the retrospective design of the study. Notably, infarct core volume was neither assessed nor incorporated into the statistical models. Infarct core volume has the potential to influence both baseline severity and clinical outcome; therefore, its omission may partially confound the observed associations. Notably, our inability to analyze thrombi from failed thrombectomy procedures also represents a significant selection bias, potentially overestimating NETs prevalence in recalcitrant, fibrin-rich clots. Methodologically, our quantification of NETs relied on two-dimensional immunofluorescence, which may not fully capture the complex three-dimensional heterogeneity of these structures within the thrombus, and there is currently no universally accepted gold standard for such quantification. Most importantly, the observational nature of this research, while demonstrating a strong association between high NETs burden and poor outcomes, cannot establish causality. It remains uncertain whether abundant NETs are a direct pathogenic driver of microvascular injury or merely an epiphenomenon of a more severe underlying thrombo-inflammatory state. Fourthly, the single-center data and relatively small sample size limit statistical power and the generalizability of our findings. Additionally, the predictive performance of our model was only moderate (ROC AUC: 0.644), suggesting that further optimization and external validation are warranted. Finally, we did not specifically demonstrate the presence of circulating NETs in our study to predict the potential pharmacotherapy targeting NETs after removal of thrombus in AIS patients. However, thrombus formation itself is a consequence of circulating NETs. In follow-up studies, we will further examine circulating NETs to explore their role in secondary microthrombosis and inflammation. Therefore, large-scale, prospective, multicenter validation is imperative to confirm these results and elucidate the precise causal role of NETs in ischemic stroke pathophysiology.

## Conclusion

In patients with acute ischemic stroke undergoing mechanical thrombectomy, a high content of NETs within the retrieved thrombus is independently associated with poor 90-day functional outcome. This relationship persists even after successful vessel recanalization, implicating NETs in the downstream pathophysiology of ischemia–reperfusion injury. Thrombus NETs analysis may represent a promising candidate biomarker for risk stratification and could inform the future design of targeted adjunctive therapies aimed at improving outcomes in this high-risk population.

## Data Availability

The raw data supporting the conclusions of this article will be made available by the authors, without undue reservation.
